# Outcomes following liver transplant for alcohol-associated liver disease: comparing alcohol-associated hepatitis and cirrhosis

**DOI:** 10.1097/HC9.0000000000000132

**Published:** 2023-05-04

**Authors:** Matthew Schroeder, Mark Pedersen, Jan Petrasek, Lafaine Grant

**Affiliations:** 1 Department of Internal Medicine, University of Texas Southwestern Medical Center, Dallas, Texas, USA; 2 Division of Digestive and Liver Diseases, University of Texas Southwestern Medical, Dallas, Texas, USA; 3 Division of Digestive Disease, University of Mississippi, Mississippi, USA

## Abstract

Liver transplant (LT) is a highly effective therapy for refractory severe alcohol-associated hepatitis (SAH), but optimal selection criteria remain unknown. We aim to evaluate the outcomes of patients who received LT for alcohol-associated liver disease at our center following the introduction of updated selection criteria, including the removal of the minimum sobriety requirement.

**Methods:**

Data were collected on all patients who underwent LT for alcohol-associated liver disease from January 1, 2018, to September 30, 2020. Patients were divided into SAH and cirrhosis cohorts based on disease phenotype.

**Results:**

One hundred twenty-three patients underwent LT for alcohol-associated liver disease, including 89 (72.4%) for cirrhosis and 34 (27.6%) for SAH. There was no difference in 1- (97.1 ± 2.9% vs. 97.7 ± 1.6%, *p* = 0.97) and 3-year (97.1 ± 2.9% vs. 92.4 ± 3.4%, *p* = 0.97) survival between SAH and cirrhosis cohorts. Return to alcohol use was more frequent in the SAH cohort at 1 year (29.4 ± 7.8% vs. 11.4 ± 3.4%, *p* = 0.005) and 3 years (45.1 ± 8.7% vs. 21.0 ± 6.2%, *p* = 0.005) including higher frequencies of both slips and problematic drinking. Unsuccessful alcohol use counseling (HR 3.42, 95% CI 1.12–10.5) and prior alcohol support meetings (HR 3.01, 95% CI 1.03–8.83) predicted a return to harmful alcohol use patterns in early LT recipients. Both duration of sobriety (*c*-statistic 0.32 (95% CI 0.34-0.43) and SALT score (c-statistic 0.47, 95% CI 0.34–0.60) were independently poor predictors of return to harmful drinking.

**Conclusion:**

Survival following LT was excellent in both SAH and cirrhosis cohorts. Higher rates of return to alcohol use highlight the importance of further individualized refinement of selection criteria and improved support following LT.

## Graphical abstract

**Figure:**
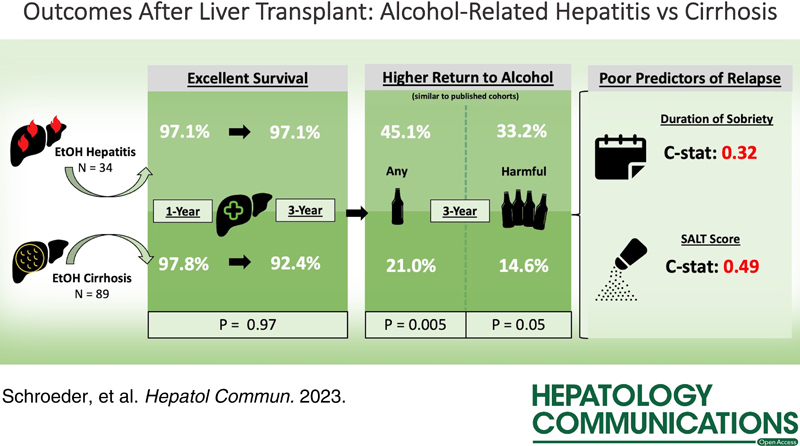

## INTRODUCTION

Over the past decade, early liver transplant (LT), defined as surgery before at least 6 months of sobriety, has emerged as an increasingly accepted therapy for severe alcohol-associated hepatitis (SAH). Promising findings from an initial randomized pilot study^1^ have been replicated in both large single-center^2^ and multicenter cohort studies.^3^ Similar findings were reproduced in a recent analysis of United Network for Organ Sharing data demonstrating graft survival following early LT of 91.7% and 81.9% at 1 and 5 years, respectively.^4^ While the number of centers performing LT for SAH has increased in recent years,^5^,^6^ practices vary by center, region, and country.^4^,^7^–^9^

Concerns persist regarding the return to harmful alcohol use after transplant. While most patients maintain their sobriety after transplant, both early and heavy alcohol use after LT have been shown to significantly impact survival.^10^ The dissolution of the 6-month rule has compelled programs to develop increasingly nuanced ways of evaluating an individual patient’s risk for returning to problematic alcohol use patterns after transplant. Risk factors for return to problematic drinking were historically evaluated separately in patients with decompensated cirrhosis with prolonged sobriety and in patients with SAH with a short duration of sobriety.

In our study, we evaluate posttransplant outcomes of patients who underwent LT for any alcohol-associated liver disease (ALD) from a single tertiary referral center following the introduction of an updated pre-LT evaluation protocol, which included the removal of a minimum pre-LT sobriety requirement. This real-world data includes a comparison of the outcomes and risk factors for return to problematic drinking in patients who underwent LT for alcohol-associated cirrhosis and SAH.

## METHODS

### Patient selection

All patients greater than 18 years of age were included in the analysis if they underwent LT for ALD after January 1, 2018 through September 1, 2020, following the introduction of an updated transplant protocol for patients with ALD at our center. This reflects a referral period from June 30, 2016, to September 1, 2020. Data were collected at the time of the initial transplant evaluation.

Patients were retrospectively reviewed for inclusion into either SAH or alcohol-associated cirrhosis cohorts. Patients were included in the SAH group who had (1) regular heavy alcohol use (> 40 g daily or > 280 g weekly on average for women and > 60 g daily or > 420 g weekly for on average men for the last 6 months), (2) less than 8 weeks of sobriety prior to the onset of jaundice (bilirubin >3 mg/dl), (3) characteristic elevations in transaminases (AST ≥50 IU/mL but < 500 IU/mL, and AST/ALT ratio ≥ 1.5), (4) liver biopsy showing alcohol-associated hepatitis or imaging showing increased echogenicity, (5) no prior history of chronic liver disease, and (6) discriminant function greater than 32. Patients were included in the cirrhosis group who did not meet the criteria for the SAH group but otherwise had evidence of end-stage liver disease, a history of regular, heavy alcohol use (as defined above), and a lack of alternate diagnosis as determined by the primary hepatologist. All patients underwent testing for other causes of chronic liver disease, including viral hepatitis, as part of routine care during their transplant evaluation.

### Transplant evaluation

All patients referred to LT for ALD underwent a standardized medical and psychosocial evaluation prior to review by the transplant committee, including evaluations by transplant hepatology, transplant surgery, case management, and social work. All patients with a diagnosis of SAH were required to undergo evaluation by addiction psychology. For other patients, evaluations by addiction psychology and psychiatry were made at the discretion of the primary hepatologist. Decisions for waitlisting were determined on a case-by-case basis by a consensus vote of the transplant committee. A commitment to lifelong abstinence was required of all patients prior to consideration. Patients deferred or denied upon initial review by the transplant committee were eligible for repeat consideration following adherence to the recommended abstinence support program. As part of the updated 2018 protocol, no minimal sobriety duration was required for patients with ALD prior to waitlisting.

### Posttransplant follow-up

Following LT, transplant recipients were seen routinely by a hepatologist on a standardized schedule. At each follow-up visit, patients underwent routine history and laboratory evaluation to monitor graft function and return to alcohol use. Rejection was defined by liver biopsy, with typical histologic features or elevated liver chemistries that resolved with the empiric escalation of immunosuppression. Follow-up interval was determined by the patient's last contact with outpatient monitoring.

Return to alcohol (“relapse”) was defined as any documented alcohol use after transplant recorded by either self-report or laboratory screening. At each visit, transplant providers inquired about alcohol use, participation in structured sobriety support programs, and follow-up with an addiction specialist. Referral to addiction psychiatry was made at the discretion of the provider. Utilization of laboratory markers of alcohol use was made at the discretion of each provider. Detection of elevated values of phosphatidylethanol or ethyl glucuronide above the manufacturer’s defined thresholds was recorded as a positive screen.

Alcohol use patterns following LT were defined similarly to the prior study.^3^ Short interval relapse (“slip”) was defined as any alcohol use for a duration of less than 100 days. Prolonged alcohol use was defined as consistent alcohol use greater than 100 days. Frequent alcohol use was defined as any alcohol use 4 or more days per week for at least 1 month. Binge alcohol use was defined as 6 or more drinks per day in men or 4 or more drinks per day in women. Problematic alcohol use was defined as any history of prolonged, frequent, or binge alcohol use patterns after a liver transplant.

### Statistical analysis

Continuous variables were compared with the student *T* test, and categorical variables were compared with Fisher exact test. Cox proportional hazard modelling was used for return to harmful alcohol use to calculate adjusted HR (aHR) and 95% confidence intervals (CI) for each risk factor. Patients were censored at the time of return to harmful drinking, the time of death, or the last visit if alive at the end of the study period. For both Cox regression models, variables with a *p* ≤ 0.20 on univariate regression were incorporated into a multivariable model with stepwise backward elimination of variables with *p* > 0.10. Otherwise, *p* < 0.05 was considered significant. The effects of multicollinearity in the model were evaluated with the variance inflation factor. Overall survival and return to harmful drinking were evaluated by the Kaplan-Meier method with the log-rank test.

SALT (Sustained Alcohol Use Posttransplant) scores were retrospectively calculated, with components including > 10 drinks per day at the time of hospitalization (+4 points), multiple prior rehabilitation attempts (+4), prior alcohol-associated legal issues (+2 points), and prior non-marijuana illicit substance use (+1).^11^

The diagnostic accuracy of the duration of sobriety and the SALT score was evaluated using receiver operating characteristic (ROC) curve analysis, using the AUROC to calculate the *c*-statistic and its 95% CI. SPSS software (Statistical Product and Services Solutions, version 27, Chicago, IL, USA) was used for statistical analysis.

## RESULTS

### Cohort selection

**FIGURE 1:**
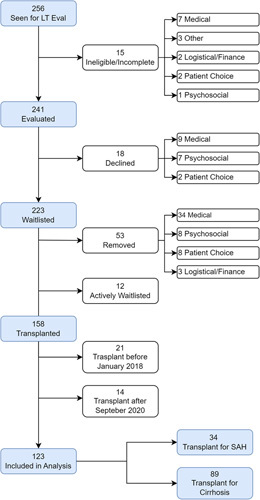
Outcomes of patients referred to liver transplant evaluation for alcohol-associated liver disease.

From June 30, 2016, to September 1, 2020, a total of 256 patients with ALD were seen at our center following a referral for LT (Figure 1). Selected patients were first seen by a transplant hepatologist at our center prior to referral for transplant evaluation if deemed appropriate by updated selection criteria. Fifteen (5.6%) patients were ineligible and did not undergo formal LT evaluation due to medical (n = 7), logistical/financial (n = 2), personal choice (n = 2), psychosocial (n = 1), or miscellaneous (n = 3) related factors. Of patients evaluated by the transplant committee, 223 of 241 (92.5%) were waitlisted for LT.

Following evaluation, the primary reasons to decline waitlisting were secondary to medical (n = 9) and psychosocial (n = 7) contraindications. Of patients waitlisted, 53 (23.8%) patients were later removed due to medical illness or death (n = 34), psychosocial (n = 8), patient choice (n = 8), and logistical/finance (n = 3). Twelve patients remained active on the waitlist at the end of the study period. In total, 158 (61.7%) patients underwent liver transplantation. After the exclusion of 21 patients transplanted before January 2018 and 14 transplanted after September 2020, there were 123 (48%) patients with ALD who underwent LT during the study period and were included in the analyses.

### Pretransplant demographics

During the study period, 89 (72.4%) patients with cirrhosis and 34 (27.6%) with SAH underwent liver transplants for ALD (Table 1). Four patients with SAH were not immediately listed but reconsidered after a period of 4 weeks with documented negative alcohol testing and participation in either addiction psychiatry or mutual aid group. Compared with patients with cirrhosis, those with SAH were younger (42.7 vs. 56.9 y, *p* < 0.001) and had higher disease severity at the time of waitlisting (model of end-stage liver disease sodium 36.0 vs. 21.7, *p* < 0.001) and transplant (model of end-stage liver disease sodium 36.5 vs. 27.5, *p* < 0.001). There was a higher proportion of female patients in the SAH cohort (50.0% vs. 19.1%, *p* = 0.001). There was no significant difference in the ethnic composition of the 2 groups (*p* = 0.06). Prior to transplant, 15 (42.9%) patients with SAH received systemic steroids with a median Lille score of 0.804 (interquartile range 0.309 – 0.980). The most common contraindications to steroids included renal failure (65.0%), infection (40.0%), and acute gastrointestinal bleeding (10%), noting that some patients had more than 1 contraindication.

**TABLE 1:** Pretransplant characteristics of patients with SAH and cirrhosis

	SAH (N = 34)	Cirrhosis (N = 89)	*p* value
Age at transplant	42.7 ± 10.2	56.9 ± 8.4	<0.001
Male	17/34 (50.0)	72/89 (80.9)	0.001
Ethnicity (%)	—	—	0.06
White	27/34 (79.4)	52/89 (58.4)	—
Asian	1/34 (2.9)	1/89 (1.1)	—
Black	0/34 (0.0)	2/89 (2.2)	—
Hispanic or Latino	4/34 (11.8)	32/89 (36.0)	—
Native American or Native Alaskan	2/34 (5.9)	2/89 (2.2)	—
Alcohol U/Day	8.8 ± 4.7	9.0 ± 6.3	0.79
Duration of Prior Sobriety (mo)	1.1 ± 1.2	33.2 ± 44.8	< 0.001
Duration of Heavy Alcohol Use (y)	12.5 ± 7.0	22.0 ± 10.1	< 0.001
MELD-Na at listing	36.0 ± 4.5	21.7 ± 8.1	< 0.001
MELD-Na at transplant	36.5 ± 5.1	27.5 ± 6.0	< 0.001
Time on transplant list (D)	8.2 ± 8.9	165.4 ± 214.3	<0.001
Choice of alcohol (%)			
Beer	13/34 (38.2)	48/89 (53.9)	0.16
Liquor	24/34 (70.6)	44/89 (49.4)	0.04
Wine	14/34 (41.2)	19/89 (21.3)	0.04
SIPAT Score	17.4 ± 8.8	12.3 ± 8.4	<0.001
SALT Score	2.0 ± 3.0	1.8 ± 2.4	0.66
Social Risk Factors (%)			
Legal citations	10/34 (29.4)	31/89 (34.8)	0.67
Family history of alcohol abuse	14/34 (41.2)	29/89 (32.6)	0.40
Illicit drug use history	6/34 (17.6)	19/89 (21.3)	0.80
Employed	28/34 (82.4)	27/89 (30.3)	<0.001
Prior alcohol rehabilitation	9/34 (26.4)	9/89 (10.1)	0.04
Prior counseling	5/34 (14.7)	16/89 (18.0)	0.792
Addiction psychiatry	7/34 (20.6)	20/89 (22.5)	1.00
Pharmacologic support	1/34 (2.9)	2/89 (2.2)	1.00
Prior meetings	7/34 (20.6)	15/89 (16.9)	0.61
Household companion	26/34 (76.5)	66/89 (74.2)	1.00
Adult children	10/34 (29.4)	58/89 (65.2)	<0.001
Young children	17/34 (50.0)	17/89 (19.1)	0.001
Insurance (%)	—	—	<0.001
Private	31/34 (91.2)	50/89 (56.2)	—
Medicare	1/34 (2.9)	31/89 (34.8)	—
Medicaid	2/34 (5.9)	8/89 (9.0)	—

Abbreviation: MELD-Na, model of end-stage liver disease sodium.

Daily alcohol use was similar between the 2 cohorts; however, the SAH group had a shorter duration of heavy alcohol use (12.5 vs. 22.0 y, *p* < 0.001), shorter duration of sobriety prior to transplant (1.1 vs. 33.2 mo), and shorter time on transplant waitlist (8.2 vs. 165.4 d, *p* < 0.001). Ten (11.2%) patients in the cirrhosis cohort had a duration of sobriety less than 6 months before transplant but did not otherwise meet the criteria for the SAH cohort. Pre-LT psychosocial evaluation revealed no difference in the frequency of legal citations, prior illicit drug use, family history of substance abuse, or the presence of a household companion. Patients who underwent transplant for SAH were more likely to be recently employed (82.4% vs. 30.3%, *p* < 0.001), have children living in the home (50.0% vs. 19.1%, *p* = 0.001), have private insurance (91.2% vs. 56.2% *p* = 0.001), and to have failed a prior alcohol rehabilitation program (26.4% vs. 10.1%, *p* = 0.04). Patients who underwent LT for cirrhosis had lower Stanford Integrated Psychosocial Assessment for Transplant scores (12.3 vs. 17.4, *p* < £0.001) compared with those with SAH.

### Posttransplant outcomes

**FIGURE 2:**
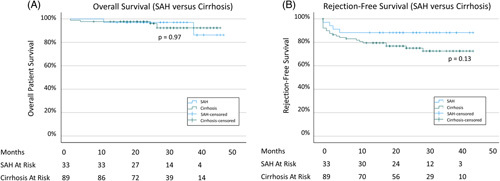
Survival analysis by SAH versus cirrhosis for (A) overall survival and (B) rejection-free survival. Abbreviation: SAH, severe alcohol-associated hepatitis.

Median follow-up for the cohort was 28 months, with a similar duration for the 2 groups. Overall survival at 1- and 3 years was high for both groups, with no significant difference between the SAH and cirrhosis cohorts at 1 year (97.1 ± 2.9% vs. 97.8 ± 1.6%, *p* = 0.97) or 3 years (97.1 ± 2.9% vs. 92.4 ± 3.4%, *p* = 0.97), (Table 2), Figure 2A. Rates of rejection were also similar between the 2 groups at 1 year (11.8 ± 5.5% vs. 20.5 ± 4.3%, *p* = 0.13) and 3 years (11.8 ± 7.6% vs. 23.2 ± 5.2%, *p* = 0.13), Figure 2B.

**TABLE 2:** Post-transplant outcomes

	SAH (N = 34)	Cirrhosis (N = 89)	*p* value
Posttransplant	—	—	0.97
1-year survival	97.1 ± 2.9	97.8 ± 1.6	—
3-year survival	97.1 ± 2.9	92.4 ± 3.4	—
Rejection	—	—	0.13
1-year rejection	11.8 ± 5.5	20.5 ± 4.3	—
3-year rejection	11.8 ± 7.6	23.2 ± 5.2	—
Relapse	—	—	0.005
1-year relapse	29.4 ± 7.8	11.4 ± 3.4	—
3-year relapse	45.1 ± 8.7	21.0 ± 6.2	—
Problematic Relapse	—	—	0.05
1-year problematic relapse	26.5 ± 7.6	11.4 ± 4.0	—
3-year problematic relapse	33.2 ± 9.7	14.6 ± 5.0	—
Slips	—	—	0.08
1-year slips	2.9 ± 2.9	0.0 ± 0.0	—
3-year slips	12.2 ± 5.7	2.8 ± 2.0	—
Laboratory alcohol screenings (mean count)			
Any alcohol screen	3.4 ± 2.4	1.3 ± 1.4	<0.001
Phosphatidylethanol or Ethyl			
Gluconuride Testing	2.4 ± 1.9	0.7 ± 1.4	<0.001
Method relapse detect			
Interview	2/15	6/17	0.87
Laboratory markers	12/15	10/17	0.01
Elevated transaminases	6/15	4/17	0.03
Addiction psychiatry (%)	22/34 (64.7)	18/89 (20.2)	<0.001
Pharmacologic support(%)	6/34 (17.6)	4/89 (4.5)	0.03
Addiction support meetings(%)	13/34 (38.2)	8/89 (9.0)	<0.001

* All survival estimateiks are reported with SE.

**FIGURE 3:**
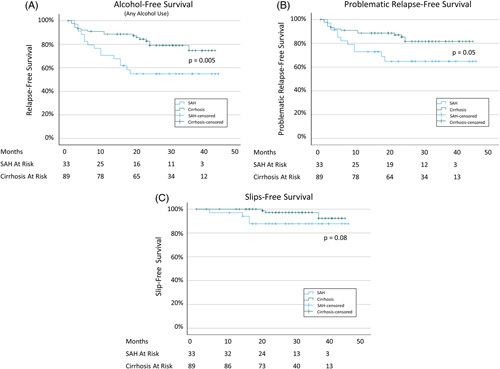
Relapse-free survival analysis by SAH versus cirrhosis for (A) all relapse, (B) problematic relapse, and (B) slips. Abbreviation: SAH, severe alcohol-associated hepatitis.

Return to drinking was higher in the SAH cohort at 1 year (29.4 ± 7.8% vs. 11.4 ± 3.4%, *p* = 0.005) and 3 year (45.1 ± 8.7% vs. 21.0 ± 6.2%, *p* = 0.005). This was both increased slips (year 1: 2.9 ± 2.9% vs. 0.0%, *p* = 0.08; year 3: 12.2 ± 5.7% vs. 2.8 ± 2.0% *p* = 0.08) and problematic drinking (year 1: 26.5 ± 7.6% vs. 11.4 ± 4.0%, *p* = 0.05; year 3: 33.2 ± 9.7% vs. 14.6 ± 5.0%, *p* = 0.05) Figure 3A. Of the 4 patients that were reconsidered at 4 weeks, only 1 has had prolonged, ongoing alcohol relapse.

Laboratory markers of alcohol use were the most common method for the detection of relapse in both groups. Screening with phosphatidylethanol and ethyl gluconuride testing was more common in the SAH group (3.4 ± 2.4 vs. 0.7 ± 1.4, *p* < 0.001). Patients in the SAH group were more likely to be seen by addiction psychiatry (64.7% vs. 20.2%, *p* < 0.001), receive pharmacologic support for alcohol cravings (17.6% vs. 4.5%, *p* 0.03), and attend structured addiction support meetings (38.2% vs. 9.0%, *p* < 0.001) following transplant. Nonetheless, only 40 patients (32.5%) participated in addiction psychiatry. Of these, 22 were patients who had already returned to alcohol. Of the patients who returned to problematic drinking, only 8 in the SAH cohort and 14 in the cirrhosis cohort returned to prolonged drinking. Of the 7 deaths in this study, 3 (2 in the SAH cohort and 1 in the cirrhosis cohort) were related to graft failure from a combination of relapse and chronic rejection.

### Factors predicting a return to harmful drinking

Multivariable Cox regression analysis identified duration of prior sobriety in months (aHR 0.96, 95% CI 0.93–0.99) and history of unsuccessful alcohol use counseling (aHR 4.00, 95% CI 1.80–8.92) as pre-LT factors that predict return to harmful use of alcohol after LT (Table 3). For patients who received early LT (< 6 mo documented sobriety), unsuccessful alcohol use counseling (aHR 3.42, 95% CI 1.12– 10.5) and prior attendance of alcohol support meetings (aHR 3.01, 95% CI 1.03 – 8.83) predicted a return to harmful alcohol use patterns. VIF for all variables in the final model was ≤ 2.0, indicating minimal collinearity. Duration of prior sobriety performed poorly alone in predicting sobriety post-LT (excluding slips), with a *c*-statistic of 0.32 (95% CI 0.34–0.43). A cutoff of 6 months of sobriety had a sensitivity of 72% and specificity of 44% in predicting return to harmful drinking. The SALT score was not useful in our population to predict harmful drinking with a *c*-statistic of 0.47 (95% CI 0.34–0.60). An increased SALT score ( > 5) had a sensitivity of 18.5% and a specificity of 84.4% in predicting return to harmful patterns of alcohol use in our population.

**TABLE 3:** Cox regression analysis for factors predicting return to harmful alcohol use

	Entire population		Sobriety < 6 mo	
	Univariable	Multivariable	Univariable	Multivariable
Pretransplant variables	HR (95% CI)	aHR (95% CI)	HR (95% CI)	aHR (95% CI)
Male sex	1.19 (0.48–2.99)	—	1.32 (0.50–3.5)	—
Age	0.96 (0.93–0.99)	—	0.99 (0.95–1.03)	—
Race	0.95 (0.72–0.13)	—	1.23 (0.85–1.78)	—
Duration of prior sobriety	0.96 (0.93–0.99)	0.97 (0.93–0.99)	—	—
Alcohol-associated hepatitis	0.43 (0.20–0.97)	—	0.631 (0.25–1.59)	—
Number of drinks per day	0.99 (0.93–1.07)	—	1.04 (0.94–1.15)	—
SIPAT Score	1.02 (0.99–1.07)	—	1.01 (0.96–1.07)	—
Prior alcohol rehabilitation	3.46 (1.45–8.06)	—	2.55 (0.99–6.62)	—
Legal citations	1.32 (0.59–2.95)	—	2.17 (0.86–5.49)	—
Family history of alcohol abuse	2.44 (1.11–5.36)	—	2.40 (0.93–6.19)	—
History of illicit drug use	0.72 (0.25–2.09)	—	0.53 (0.12–2.30)	—
Psychiatry history	1.06 (0.40–2.81)	—	1.48 (0.49–4.51)	—
Employed	1.69 (0.76–3.72)	—	0.81 (0.31–2.17)	—
Addiction psychiatry	1.93 (0.83–4.45)	—	1.33 (0.52–3.44)	—
Counseling	4.30 (1.92–9.59)	4.00 (1.80–8.92)	6.00 (2.23–15.7)	3.42 (1.12–10.5)
Sobriety support meetings	3.56 (1.60–7.93)	2.25 ( 0.93–5.42)	4.85 (1.91–12.3)	3.01 (1.03–8.83)

Abbreviation: SIPAT, Stanford Integrated Psychosocial Assessment for Transplant.

## DISCUSSION

This study contributes to the growing body of evidence supporting the expanded use of LT for select patients with SAH. In our patient population, the one-year survival was 97.1% for patients with SAH and 97.7% for patients with cirrhosis. This is similar to the 93.6% 1-year survival seen nationally in liver transplantation for any etiology^12^ and the 94% 1-year survival from the American Consortium of Early Liver Transplantation for Alcohol-associated Hepatitis (ACCELERATE-AH).^3^ Survival remained excellent at 3 years at 97.1% in the SAH group and 92.4% in the cirrhosis group.

Cumulative return to alcohol use was higher in the SAH group than the cirrhosis group at 3 years (45.1 ± 8.7% vs. 21.0 ± 6.2%, *p* = 0.047). Approximately one-quarter of relapses in the SAH group were short interval slips. These rates of return to alcohol are marginally higher than recent studies, which have reported return to alcohol use after early LT in the ranges of 20–30% and 30–40% at 1- and 3-years post-LT, respectively.^2^,^10^,^13^

Prior studies comparing rates of relapse-free survival between SAH and cirrhosis groups have suggested these rates were similar.^2^,^14^ These studies, however, reported only relapse-free survival at 1 year and not beyond. Our study echoes the findings of a recent prospective study from Louvet et al, demonstrating increased rates of return to harmful alcohol in the early LT group (22% vs. 5%, 95% CI 5.8–27.6) when following patients out to 2 years.^13^ Even so, less than a fifth of the patients in our study returned to prolonged drinking, highlighting the need for early relapse detection and robust, easy-to-access sobriety counseling, and support groups to help patients who do.

In the total population, factors that predicted return to harmful drinking included prior duration of sobriety and prior failed alcohol counseling. These predicted a return to harmful alcohol use in all recipients with ALD. In patients with early LT, failure of counseling and prior attendance at structured support meetings was predictive in our cohort. These factors were similar to those described by Lee and colleagues, which included heavy alcohol use prior to hospitalization, multiple prior rehabilitation attempts, alcohol-associated legal citations, and prior illicit substance abuse.^11^ In our study, while the duration of pre-LT sobriety did predict sustained post-LT sobriety, setting a hard time cutoff was prone to misclassification with an AUROC of 0.32. A 6-month cutoff would have incorrectly classified 28 patients. The previously published SALT score was also not particularly helpful in our patient population.

When compared with prior studies, the rate of listing patients with alcohol-associated liver disease at 92% is notably high. Several factors unique to our population may explain this. First, the population contains many patients with standard cirrhosis with a prolonged duration of sobriety. Second, significant prescreening of patients occurs, which is not captured, through hepatology consultations of admitted patients or careful discussion of potential transfer with referring providers. Neither of these cohorts would have generated a liver transplant referral to be captured in the denominator.

Also different is the frequency of psychosocial risk factors such as alcohol-associated legal citations, prior failed rehabilitation, and family history of alcohol use disorder.^3^ While this is acknowledged, our population represents the full spectrum of alcohol-associated transplant recipients. This includes patients transplanted very early for SAH and those who underwent individual and/or group therapy and then were reconsidered. All patients in our study who had these higher risk features were required to participate in group therapy (such as Alcohol-associated Anonymous or SMART recovery), addiction psychiatry, or therapy with a licensed alcohol counselor for a period of time. Hence, the focus is not only on risk identification but also on risk mitigation: matching the patient’s risk of return to harmful drinking and acuity of illness with sobriety-promoting activities that foster success. With only a third of patients participating in addiction psychiatry after LT, there is an opportunity to standardize and more proactively promote risk mitigation.

The study has several limitations. The study is retrospective in design, leading to potential measurement bias and unmeasured confounders. Baseline characteristics between the cirrhosis and SAH groups were different, reflecting observed differences in the 2 populations in clinical practice. In addition, those in the SAH cohort had a higher rate of private insurance funding. This may be partly explained by the younger age of the cohort compared with those with cirrhosis who were older and more likely to have Medicare funding. Incidentally, our center is located in a state where Medicaid was not expanded and where Medicaid historically has contracted with only a single liver transplant center.

Providers were not blinded to transplant indication at follow-up visits, which may have resulted in the heterogenous application of both screening and relapse prevention interventions. Lower than expected rates of relapse in the cirrhosis group may suggest the missed incidence of return to alcohol use, as suggested by the lower rate of phosphatidylethanol and ethyl gluconuride measurements seen in this group. However, these were presumably short-lived and mild, avoiding significant allograft dysfunction expected with problematic drinking. Finally, the most effective measures of reporting and quantifying return to alcohol use after LT have yet to be determined. We opted to combine relapse patterns of prolonged, frequent, and binge drinking into a single composite outcome for analysis, given the low event rate of each specific pattern. Slips were excluded as their short duration is unlikely to affect allograft function. Analysis of binary hazard-free survival following a single episode of problematic drinking may overlook the lifelong nature of alcohol use disorder. Measures of cumulative alcohol exposure and duration of heavy drinking may better quantify harmful patterns of alcohol use and better reflect the risks of graft dysfunction.^13^,^15^,^16^

In conclusion, our study further supports the use of LT for SAH as a life-saving option for those patients unresponsive to medical therapy. Though alcohol relapse was higher in the SAH cohort, 1- and 3-year survival remained excellent. Ongoing refinement of alcohol selection criteria is needed, though it may vary based on the biases at each particular program.
